# Symptoms and health‐related quality of life 5 years after catheter ablation of atrial fibrillation

**DOI:** 10.1002/clc.23752

**Published:** 2021-12-16

**Authors:** Ulla Walfridsson, Anders Hassel Jönsson, Lars O. Karlsson, Ioan Liuba, Henrik Almroth, Emma Sandgren, Håkan Walfridsson, Emmanouil Charitakis

**Affiliations:** ^1^ Department of Cardiology University Hospital Linköping Sweden; ^2^ Department of Medical and Health Sciences Linköping University Linköping Sweden

**Keywords:** atrial fibrillation, disease‐specific questionnaire, health‐related quality of life, long‐term follow‐up, symptoms

## Abstract

**Objectives:**

To investigate the effect of catheter ablation (CA) on symptoms and health‐related quality of life (HRQoL) after 5 years, and analyze predictors of recurrence of symptoms.

**Background:**

The primary indication for CA of atrial fibrillation (AF) is to reduce symptoms and improve HRQoL where long‐term follow‐up are sparse.

**Methods:**

In this observational, long‐term, single‐center study, patients were recruited from Linköping University Hospital, Sweden. They were aged ≥18 years and had been referred for CA from November 2011 until June 2019. Arrhythmia‐specific symptoms and HRQoL were assessed by patient‐reported outcome measures (PROMs) with the Arrhythmia‐Specific questionnaire in Tachycardia and Arrhythmia (ASTA).

**Results:**

In the study were 1521 patients, 69% men, mean age 62 years. At baseline, 87% of the patients and at the 5‐year follow‐up 80% of those eligible filled out the ASTA questionnaire. At follow‐up, 50% reported freedom from symptoms, 18% had >50% symptom reduction, 14% had a minor reduction, while 18% reported no effect or a worsening of symptoms. Factors predicting symptoms were female gender (hazard ratio [HR]: 1.8; 1.2–2.8), body mass index ≥ 35 (HR: 3.9; 1.6–9.8), and ischemic heart disease (IHD) (HR: 2.6; 1.2–5.9). After 5 years, breathlessness during activity, weakness/fatigue, and tiredness were still the most common symptoms; regarding HRQoL they were impaired physical ability and deteriorated life situation.

**Conclusions and Clinical Implications:**

This clinical cohort of patients with AF evaluated through PROMs showed that CA had long‐lasting effects on symptoms and HRQoL and that the use of PROMs in clinical routines was feasible. Factors predicting symptoms after CA were female gender, IHD, and obesity, an important reminder to encourage lifestyle management.

**Abbreviations**
AF: atrial fibrillation
ASTA: Arrhythmia‐Specific questionnaire in Tachycardia and Arrhythmia
BMI: body mass index
CA: catheter ablation
ESC: European Society of Cardiology
HRQoL: health‐related quality of life
IHD: ischemic heart disease
LVEF: left ventricle ejection fraction
PROMs: patient‐reported outcome measures
TIA: transient ischemic attack

## 1 INTRODUCTION

Treatment outcomes assessed by patient‐reported outcome measures (PROMs) need to be addressed in patients with atrial fibrillation (AF) and should be included in clinical routines in connection with catheter ablation (CA).^1^, ^2^, ^3^ AF‐related symptoms can be graded either with the physician‐assessed European Heart Rhythm Association score or with PROMs.^4^ According to the European Society of Cardiology (ESC) guidelines, treatment with CA is indicated in symptomatic AF patients with negatively affected daily life. Importantly, guidelines from 2020 highlight the use of PROMs and recommend disease‐specific evaluations in clinical routines.^5^, ^6^ One such tool for assessment is the Arrhythmia‐Specific questionnaire in Tachycardia and Arrhythmia (ASTA), developed and validated in a Swedish patient population with AF, supraventricular and ventricular arrhythmias, and which assesses symptom burden and health‐related quality of life (HRQoL).^7^, ^8^

Even though the primary indication for CA in patients with AF is symptom relief, most studies report results based upon rhythm rather than symptoms and effects on HRQoL, and the follow‐up is seldom long‐term. The aim of this paper was to investigate the effect of CA on symptoms and HRQoL and to analyze possible predictors of remaining symptoms 5 years after CA.

## 2 METHODS

### 2.1 Study design

This was an observational, single‐center, long‐term cohort study in a Swedish patient population treated with CA of AF at the University Hospital, Linköping, Sweden, between November 2011 and June 2019.

### 2.2 Patients and follow‐up

Consecutive patients referred for CA of AF were asked to complete the ASTA questionnaire before and 5 years after CA, as part of the clinical routine. Patients received written instructions approximately 2 weeks before the CA on how to access and fill out the ASTA questionnaire. At the time of follow‐up, the patients received written instructions on how to access the questionnaire. Those not responding within one month received a reminder. Patients included were those aged ≥18 years with sufficient knowledge of the Swedish language, capable of independently filling out the questionnaire, and willing to participate.

### 2.3 PROMs with the arrhythmia‐specific ASTA questionnaire

The first part of the ASTA questionnaire evaluates the latest episode of arrhythmia and current medication. The second part assesses symptom burden with a nine‐item symptom scale (ASTA symptom scale) with the response alternatives: “No (0), Yes, to a certain extent (1), Yes, quite a lot (2) or Yes, a lot (3).” There are also questions regarding the experiences of arrhythmia (palpitations) and on whether the respondent has ever fainted or come close to fainting. Part three assesses HRQoL with a 13‐item scale, with the same four‐point response scale (ASTA HRQoL scale). The ASTA HRQoL scale is divided into a seven‐item physical subscale and a six‐item mental subscale. Scale scores are calculated where values range from 0 to 100. Higher scores reflect a higher symptom burden and a worse effect on HRQoL due to arrhythmia. When patients answer “No, I have no arrhythmia” at follow‐up the recommended scoring for the ASTA scales is zero^7^, ^8^, ^9^ (Supporting Information 1).

### 2.4 Ethical considerations

The Regional Ethical Review Board at the Faculty of Health Sciences, Linköping, Sweden approved the extraction and publication of the clinical data (DNR 2016‐349‐31). The study complies with the Declaration of Helsinki.^10^ This evaluation is a part of the clinical routine, and oral informed consent was considered adequate.

### 2.5 Statistical analysis

For baseline data, continuous variables were expressed as means and standard deviation (±SD). Categorical data were reported as counts with percentages within brackets. The Kolmogorov–Smirnov test was used to test the normality of the samples. A *χ* ^2^ test was used for comparison over time of the presence of symptoms and HRQoL items.

**Figure 1:**
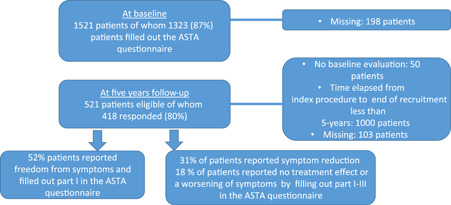
Flow chart: Five years follow‐up with the Arrhythmia‐Specific Symptoms in Tachycardia and Arrhythmia (ASTA) questionnaire in patients with atrial fibrillation treated with catheter ablation

The primary endpoint was analyzed via a mixed linear model to deal with missing data. The ASTA scale scores were used as dependent variables in different mixed model analyses. Time (baseline, and 5‐year follow‐up) was used as a repeated variable. It is important to note that this statistical method enables comparisons between one baseline group (1521 patients) and one 5‐year follow‐up group (521 patients), that is, some patients filled out the ASTA questionnaire both before ablation and at the 5‐year follow‐up, some only answered at baseline since they had not yet reached 5 years after ablation, while some filled out the questionnaire only at the 5‐year follow‐up but their data at baseline was missing. This is presented in a flow chart and in the results (Figure 1). The unstructured repeated covariance type was chosen and age ≥75 years, gender and body mass index (BMI) ≥ 35 kg/m^2^ were used as fixed factors, with the patient indicator as a random intercept. The analyses were adjusted for covariates: number of CAs per patient, ischemic heart disease (IHD), hypertension, previous stroke or transient ischemic attack (TIA), left ventricle ejection fraction (LVEF) < 50%, and the type of AF (paroxysmal or persistent).

Multiple logarithmic regression analysis was performed to determine the predictors of the persistence of symptoms. Age ≥ 75, gender, BMI ≥ 35 kg/m^2^, absence of IHD, hypertension, stroke or TIA, LVEF < 50%, type of AF, and the type of ablation catheter were used as independent predictors of symptoms 5 years after the first CA, as well as the efficacy of CA.

The models were fitted by an enter method, where all variables were entered into the original model. Variables with *p* values of >.1 were thereafter removed.

The internal consistency and homogeneity in the ASTA scales were assessed by Cronbach's *α* values and considered satisfactory when exceeding the limit of .7.^11^

All reported *p* values were two‐sided and a *p*‐value of less than .05 was considered statistically significant. The analyses were performed using the SPSS 24.0 (SPSS).

## 3 RESULTS

### 3.1 Patients

At baseline, 1323 patients out of 1521 filled out the ASTA questionnaire; 69% were men, with a mean age of 62 years (±10.18). One thousand patients were not eligible for the 5‐year follow‐up, as the time that had elapsed from the index procedure to the end of recruitment was shorter than 5 years. Finally, 521 patients were eligible for the 5‐year follow‐up. All patients received the ASTA questionnaires and 418 responded (80%), of whom 185 completed all parts of the ASTA questionnaire, that is, those still experiencing symptoms in relation to arrhythmia.

There were no differences in background characteristics between those who responded and the nonresponders. The mean number of CA procedures per patient during the follow‐up was 1.6. The most commonly used antiarrhythmic medication was a beta‐blocker, followed by amiodarone, and Class IC, mostly flecainide. The most common concomitant disease was hypertension (Table 1).

**Table 1:** Patients demographics and characteristics

Patients	Total	Responders, *N* (%)
Patients at baseline	1521	1323 (87%)
Eligible at 5‐year follow‐up	521	418 (80%)
Age at baseline (mean/±SD)	62 (±10.18)	
Age at 5‐year follow‐up (mean/±SD)	67 (±9.77)	
Gender %	69% men	

	Number (*N*)	Percent (%)
Atrial fibrillation type		
Paroxysmal AF	580	39
Persistent AF	760	51
AF and atrial tachycardia	119	8
Long‐standing persistent AF	24	2
Concomitant diseases		
Hypertension	644	42
Age ≥ 75 years	78	5
Diabetes	120	8
Stroke/TIA	102	7
HCMP	10	1
DCMP	46	3
Age ≥ 65–74 years	528	35
Vascular disease	11	0.7
IHD	132	9
PCI	83	5
CABG	30	2
Valvular surgery	42	3
Body mass index		
Women	27.7 (±5.12)	
Men	28.0 (±4.13)	
CHA_2_DS_2_ VASC points		
0	312	22.1
1	362	25.6
2	365	25.8
3	228	16.1
4	105	7.4
5	28	2.0
6	9	0.6
7	3	0.2
8	1	0.1

Medication	Baseline (*N* = 1521)	Baseline (%)	5‐year follow‐up (*N* = 418)	5‐year follow‐up (%)
Beta receptor blocker	853	62	273	65
Calcium antagonists	26	2	3	0.7
Digitalis	24	2	9	2
Class 1A	6	0.4	0	0
Class 1C	156	10	19	5
Class III	375	25	40	10
ACE inhibitors	215	16	76	18
AII‐blockers	135	10	54	13
Statins	288	19	112	27
Warfarin or NOAC	1521	100	282	67
Platelet inhibitor	24	2	14	1

Abbreviations: ACE, angiotensin‐converting enzyme; AF, atrial fibrillation; CABG, coronary artery bypass graft; DCMP, dilated cardiomyopathy; HCMP, hypertrophic cardiomyopathy; IHD, ischemic heart disease; NOAC, novel oral anticoagulant; PCI, percutaneous coronary intervention; TIA, transient ischemic attack.

### 3.2 Treatment effects of CA over time

At the 5‐year follow‐up, 52% reported freedom from AF‐related symptoms. A major reduction (>50% symptom reduction compared to baseline) was seen in 18%, and 14% had a minor reduction (10%–50%). Only 9% reported no treatment effect on symptoms and another 9% experienced a worsening of symptoms after the CA (Figure S1A symptoms and Figure S1B HRQoL).

## 4 ASTA—SYMPTOMS

### 4.1 Palpitations at baseline and at the 5‐year follow‐up

There was a change in the perception of arrhythmia at the 5‐year follow‐up in that patients more often experienced the *heart missing one or more beats*, *short episodes of arrhythmia lasting less than 1 min*, and *feeling nothing* (Figure S2A).

### 4.2 Effect of CA—ASTA symptom scale scores 5 years after the initial procedure

Patients reported a significantly lower ASTA symptom scale score 5 years after CA compared to baseline (ASTA symptom scale baseline: 37.6; 95% confidence interval [CI]: 35.9–39.4) ASTA symptom scale 5 years: 18.1 (95% CI: 14.1–22.1; *p* < .0001); Table 2) Women reported worse scores compared to men 5 years after the initial CA (ASTA symptom scale score difference between men and women at the 5‐year follow‐up: −5.6 (95% CI: −7.6 to −3.6; *p* < .0001; Table 3).

**Table 2:** Effect of catheter ablation on arrhythmia‐related symptoms and HRQoL

Scale score	Baseline (mean)	Baseline (95% CI)	5‐year follow‐up (mean)	5‐year follow‐up (95% CI)	*p*‐Value
	*N* = 1323	*N* = 1320	*N* = 418	*N* = 417	
ASTA symptoms	36.6	35.7–37.5	16	14–18	<.001
ASTA HRQoL	36.5	35.4–37.7	14.2	12.2–16.2	<.001

*Note*: Results of mixed models' analyses; ASTA symptom scale score and ASTA HRQoL scale score were used as dependent variables. Time (baseline and 5‐year follow‐up) was used as a repeated variable. Unstructured repeated covariance type was chosen, age ≥ 75, gender and BMI ≥ 35 kg/m^2^ were used as fixed factors with patient indicator as a random intercept. The analyses were adjusted for covariates: number of CAs performed per patient, the presence of ischemic heart disease, hypertension, previous stroke or TIA, Left ventricle ejection fraction < 50% and the type of AF (paroxysmal or persistent).

Abbreviations: AF, atrial fibrillation; ASTA, Arrhythmia‐Specific questionnaire in Tachycardia and Arrhythmia; BMI, body mass index; CI, confidence interval; HRQoL, health‐related quality of life; TIA, transient ischemic attack.

**Table 3:** Gender differences in symptoms and HRQoL 5 years after catheter ablation of atrial fibrillation

ASTA scale	5 years after the initial procedure (mean)	5 years after the initial procedure (95% CI)	5 years after the initial procedure (mean)	5 years after the initial procedure (95% CI)
	Women (*N* = 137)	Women (*N* = 137)	Men (*N* = 281)	Men (*N* = 281)
ASTA‐symptoms	19.2	15.6–22.8	14.4	12.1–16.8
ASTA HRQoL	15.4	12–18.8	13.6	11.1–16.1

*Note*: Results of mixed models' analyses; ASTA symptom scale score and ASTA HRQoL scale score were used as dependent variables, with gender as a fixed factor. Time (baseline and 5‐year follow‐up) was used as a repeated variable. Unstructured repeated covariance type was chosen with patient indicator as a random intercept. The analyses were adjusted for covariates: age ≥ 75, BMI ≥ 35 kg/m^2^, number of CAs performed per patient, the presence of ischemic heart disease, hypertension, previous stroke or TIA, Left ventricle ejection fraction <50% and the type of AF (paroxysmal or persistent).

Abbreviations: AF, atrial fibrillation; ASTA, Arrhythmia‐Specific questionnaire in Tachycardia and Arrhythmia; BMI, body mass index; CI, confidence interval; HRQoL, health‐related quality of life; TIA, transient ischemic attack.

Older patients (age ≥ 75 years) and younger patients (age < 75) reported the same significant improvement in the symptom scale score at the 5‐year follow‐up (*p* = .295), that is, there were no differences between the age groups.

### 4.3 Arrhythmia‐related symptoms at baseline and at the 5‐year follow‐up

The most commonly reported symptoms at baseline were *weakness/fatigue*, *breathlessness during activity* and *tiredness* and at the 5‐year follow‐up, the same symptoms were experienced but to a significantly lesser degree. *Worry/anxiety* in connection with arrhythmia was reported by 886 patients (67%) at baseline and was still present in 138 (59%) after 5 years (see Table S1).

### 4.4 Effect of catheter ablation—ASTA HRQoL scale scores 5 years after the initial procedure

The ASTA HRQoL scale score was significantly lower 5 years after CA compared to baseline (ASTA HRQoL scale score baseline: 36.7 (95% CI: 34.1–39.3), ASTA HRQoL scale score after 5 years: 13.1 (95% CI: 10.1–16.1; *p* < .0001). There were no differences in HRQoL according to gender or age. (Tables 2 and 3).

### 4.5 Arrhythmia‐related HRQoL at baseline and at the 5‐year follow‐up

The most common negative influences on HRQoL at baseline were physically related: *impaired physical ability*, *deteriorated life situation*, and *feeling unable to carry out daily activities*. At the 5‐year follow‐up, the same domains were still the most negatively affected. In those experiencing arrhythmia at follow‐up, the negative influence did not decrease significantly over time regarding *impaired physical ability* and *deteriorated life situation* (*p* = .586 and *p* = .111, respectively*). Negatively affected sexual life* was reported in 779 patients (59%) at baseline and half of the patients at the follow‐up assessment. The least common influence was being *afraid of dying* due to the arrhythmia at baseline CA and *spending less time with acquaintances* at the time of follow‐up (see Table S2).

### 4.6 Predictors of arrhythmia‐related symptoms 5 years after the initial procedure

Factors predicting the presence of symptoms 5 years after the initial CA were female gender, with an 80% increased risk of reporting symptoms (hazard ratio [HR]: 1.8; 1.2–2.8) compared to men. Patients with a BMI ≥ 35 had a fourfold higher risk of having symptoms 5 years after the initial procedure compared to those with a lower BMI (HR: 3.9; 1.6–9.8), and the presence of IHD increased the risk by almost three times (HR: 2.6; 1.2–5.9; Table 4).

**Table 4:** Factors predicting the presence of arrhythmia‐related symptoms 5 years after catheter ablation of atrial fibrillation

Predictors (*N* = 402)	HR	95% CI	*p*‐Value
Female sex	1.8	1.2–2.8	.01
IHD	2.6	1.2–5.9	.02
BMI > 35 kg/m^2^	3.9	1.6–9.8	.004

*Note*: Multiple logarithmic regression analysis was performed to determine possible predictors of the presence of symptoms 5 years after catheter ablation of atrial fibrillation. The model was fitted by an enter method where all variables were entered into the original model and then variables with probability values of .05 were removed. The predictors that turned out to be significant are presented in this table.

Abbreviations: BMI, body mass index; CI, confidence interval; IHD, ischemic heart disease.

## 5 DISCUSSION

Our main findings were that half of the patients undergoing CA for AF were free from arrhythmia‐related symptoms at the time of follow‐up, and in those still reporting symptoms, more than 80% experienced a symptom reduction after 5 years. The three significant predictors for remaining symptomatic at the time of follow‐up were female gender, obesity, and IHD.

To the best of our knowledge, this is the largest observational long‐term follow‐up study presenting real‐life data evaluated with PROMs in patients with AF undergoing CA. The study expands the knowledge about the beneficial effects of CA on disease‐related symptoms and HRQoL, and demonstrates that the use of PROMs in clinical routines is feasible, and is a practice in line with the current ESC guidelines for patients with AF.

### 5.1 Effect of catheter ablation on symptoms—comparisons over time

Altogether, more than 80% of the study participants experienced an improved arrhythmia‐related situation, consistent with the overall aim of ablation therapy in patients with AF.^5^ These data demonstrate that the positive effect of CA on symptoms is long‐lasting. A small number of patients did not improve or experienced worsening of symptoms after CA. Some of these were accepted for reablation at the time of follow‐up.

There is one specific question in the ASTA questionnaire regarding the perception of palpitations. Interestingly, the symptom pattern changed over time in patients reporting remaining symptoms after CA. This may suggest that the symptoms could be due to extra beats rather than AF, which is consistent with an overall reduction in symptom burden in those still experiencing symptoms. It is well known that there is a limited relationship between symptoms and actual heart rhythm, and this has to be considered when interpreting this finding.^12^, ^13^

Three symptoms were dominant: *breathlessness during activity*, *weakness/fatigue*, and *tiredness* but to a lesser extent after CA. The ASTA symptom scale score decreased over time, and differences were seen between genders both before and after CA. As shown earlier, women are usually more symptomatic, have a higher heart rate during AF, are less likely to undergo CA, have the worse functional capacity, and experience more anxiety than men, all of which are seen for all types of AF.^3^, ^14^, ^15^, ^16^, ^17^

Seventy‐five years of age was chosen as a cutoff for defining elderly patients, as this age relates to the risk of thromboembolic complications in the CHA_2_DS_2_ VASC scoring.^18^ Elderly patients had the same long‐term treatment benefits as younger patients; thus our data strengthen the argument that patients with AF should not be excluded from CA solely because of age. This finding is in line with a previous study with a shorter follow‐up, demonstrating a clear benefit of CA treatment for the elderly.^19^

### 5.2 Effect of catheter ablation on HRQoL—comparisons over time

HRQoL in patients with AF is influenced by typical AF‐related symptoms but also several other factors, such as worry, potential side‐effects of medication, risk of recurrence, and the need for repeat procedures after CA. Several patient‐related factors also influence HRQoL, such as age and gender as well as comorbidities common in patients with AF. Information about what arrhythmia‐specific concerns patients experience in a long‐term perspective after CA is sparse and the definition of successful treatment may vary depending on patient or healthcare provider perspectives.

On a group level, HRQoL improved significantly over time, without any differences found between genders or age groups.

Two studies included similar cohorts to the present study comparing CA to medical therapy—CABANA and CAPTAF.^20^, ^21^ In CABANA, 2204 patients were randomized, and the study focuses on data from the 12‐month follow‐up, with assessments performed with two arrhythmia‐specific PROMs (AFEQT and MAFSI). The positive improvements seen at 12 months were sustained over the 5‐year follow‐up period. The study had a high response rate, equal to the present study. In CAPTAF, 155 patients were randomized and evaluated with a generic questionnaire (SF‐36) with a focus on general health over a 12‐month period. Both studies favored treatment with CA.

At baseline the four most commonly reported items with a negative impact on HRQoL were: “*The ability to perform physical activities,”* “*deteriorated life situation,” “avoided planning for things they would like to do,”* and “*Feel unable to work, study or carry out daily activities”* and these were still the most commonly reported items with a negative impact on HRQoL. These items in the HRQoL scale were also the most frequently reported in a previously published large study on patients with AF before CA.^3^ In one study making use of a disease‐specific questionnaire and an implantable loop recorder the reduction or absence of documented AF was closely related to the reduction or elimination of AF‐related symptoms.^22^ It is reasonable to suspect that the remaining arrhythmia was the most probable reason for the observed negative impact on HRQoL still present after 5 years.

### 5.3 Predictors of arrhythmia at 5 years after the initial procedure

There were three independent predictors of negative long‐term treatment outcome: female gender, obesity (BMI ≥ 35), and IHD. Our data is in line with previous studies that demonstrate that female gender and obesity predict arrhythmia‐related symptoms in patients with AF treated with CA.^23^, ^24^, ^25^, ^26^ As discussed previously, women usually have a higher symptom burden than men and often experience a more negatively influenced HRQoL.^3^, ^17^, ^27^ However, we did not find any gender differences regarding HRQoL at follow‐up. One explanation may be an adaptation to the symptoms, that is, subjects may have found coping strategies and thus experienced less negative influence on HRQoL. Another possibility is that the heart rate during episodes slows down over time.^15^

The mean BMI in our cohort was 27.8—equal between the sexes—confirming that many patients with AF referred for CA are overweight, some being obese, which is in line with previously reported cohorts.^23^, ^28^, ^29^ Obesity is known to be a significant predictor for reduced success rate after CA and has a negative impact on HRQoL, physical health status, and symptom burden during AF episodes.^23^, ^24^, ^25^, ^26^, ^30^, ^31^ The importance of self‐management strategies with lifestyle interventions is pointed out in the guidelines and is important to consider before referral for CA.^5^, ^29^ There is solid evidence regarding the positive effects of lifestyle management, which can lead to a reduced symptom burden, absence of AF, and positive effects on comorbidities.^26^, ^28^ The third predictive factor seen was IHD, giving a threefold increased risk of having arrhythmia‐related symptoms five years after the ablation procedure. It is known that IHD in combination with AF worsens the prognosis and has been described as “double trouble.”^23^, ^24^, ^25^, ^32^

### 5.4 Take‐home messages/clinical significance in the present study

Some factors were found to predict the presence of arrhythmia‐related symptoms after 5 years among which obesity again was pointed out, confirming the importance of engaging patients in working with lifestyle interventions when needed.

The present study expands the knowledge about the beneficial effects of CA on arrhythmia‐related symptoms and daily life concerns, demonstrating that PROMs in clinical routines are feasible and add important clinical information; a practice in line with the current ESC guidelines for patients with AF.

### 5.5 Methodological considerations, strengths and limitations

This is the largest long‐term follow‐up observational study in patients with AF undergoing CA, representing the patients' real‐life situation assessed with PROMs in a clinical setting. The current study, in contrast to many other studies, includes a nonselected patient group representing a common population with AF planned for CA, with only one exclusion criterion, which was difficulties with the Swedish language.

Large‐scale clinical follow‐up is a challenge since rhythm, symptoms, and HRQoL ideally should all be evaluated long term. The follow‐up was carried out 5 years after the procedure, irrespective of whether the patient was waiting for a new evaluation or even waiting for a repeat procedure. At the 5‐year follow‐up, 80% of eligible patients responded to the ASTA questionnaire, which constitutes a very high response rate. We strived to conduct the assessments via the ASTA web version, thus avoiding missing data and making possible direct data import into the database, something that also minimizes input errors.

The aim of the study was to explore long‐term follow‐up data, seen from the patients' perspective. Data assessed was a part of clinical routine, based on PROMs without any information on actual rhythm. An electrocardiogram provides information about the rhythm “here and now” but does not capture the overall picture.

## 6 CONCLUSIONS

This large clinical cohort of patients with AF, presenting real‐life data in a 5‐year long‐term perspective with a high response rate, showed that CA has a long‐lasting positive effect on both symptoms and HRQoL and to some extent changes the perception of AF.

Half of the patients did not experience any symptoms and more than 80% of those still having symptoms experienced an improvement.

Factors predicting remaining symptoms were female gender, the presence of IHD, and obesity, the latter serving as an important reminder to encourage lifestyle management. The use of disease‐specific PROMs for long‐term follow‐up in a routine clinical setting was feasible and added important clinical information about the care of patients with AF.

## CONFLICT OF INTERESTS

The authors declare that there are no conflict of interests.

## Supporting information

Supporting information.

Supporting information.

Supporting information.

Supporting information.

Supporting information.

Supporting information.
