# Supplementary material for: Symptoms and health‐related quality of life 5 years after catheter ablation of atrial fibrillation
Source: Clin Cardiol. 2021 Dec 16;45(1):42–50. doi: 10.1002/clc.23752 (PMC8799058; doi:10.1002/clc.23752)
Supplement: Supplementary file 1 — Supporting information. [file CLC-45-42-s005.pdf]

Patient ID	Date of completion	Time of completion
		☐ Before treatment. ☐Follow-up 1 ☐Follow-up 2

The information above will be filled in by a member of health care staff

ASTA

(Arrhythmia-Specific questionnaire in Tachycardia and Arrhythmia)

Arrhythmia specific symptoms
&
Health-related quality of life in connection with heart rhythm disturbance

Living with heart rhythm disturbance (arrhythmia), affects people in various degrees. If you are going to be, or have already been treated for any type of arrhythmia, we ask you to fill in this questionnaire.

Your arrhythmia manifests itself as attacks or persistent discomfort, e.g. palpitations, atrial fibrillation, atrial flutter or frequent extra heartbeats.

Irrespective of the type of problem you are experiencing, all heart rhythm issues in this questionnaire will be termed:

Arrhythmia

The **ASTA** questionnaire is aimed at mapping out the symptoms of your arrhythmia and how they affect your life and health.

Author: Ulla Walfridsson. Dept. of Cardiology, University Hospital, Linköping, Sweden.
Do not copy or translate without permission from the author. Version ASTA v1 2012-11-27
English version v2 2015-02-11

ASTA part I

Living with arrhythmia affects people in various degrees. By answering the following questions we would like you to describe your experience. Choose the alternative that correspond the best to your situation.

Question 1

When did you last experience arrhythmia?
*(Choose **one** alternative)*

- ☐ I have persistent arrhythmia
- ☐ I have arrhythmia on and off every day
- ☐ Less than one week ago
- ☐ Less than 1 month ago
- ☐ 1 month – less than 3 months ago
- ☐ 3 months – less than 6 months ago
- ☐ 6 months – less than 12 months ago
- ☐ More than 12 months ago

Question 2

a) Are you currently on any medication?

- ☐ No
- ☐ Yes

If **”Yes”**, please indicate which medicine(s) you take on a regular basis

____

____

____

____

____

____

____

Do not copy or translate without permission from the author. Version ASTA v1 2012-11-27
English version v2 2015-02-11

- ☐ **I have arrhythmia and will complete part II and III of the questionnaire**
- ☐ **I have arrhythmia which I do not feel but I will complete part II and III of the questionnaire**
- ☐ **No, I do not have any arrhythmia and will therefore not complete part II and III of the questionnaire**

ASTA part II - Arrhythmia specific symptoms

Arrhythmia can vary in frequency, length of time and symptoms. By answering the following questions we would like you to describe your experience. Choose the alternative (s) that correspond (s) the best to your situation.

Question 1

How many times have you experienced arrhythmia during the last three months?
*(please choose **one** alternative)*

- ☐ None at all
- ☐ Less than 5 times
- ☐ Between 5 and 15 times
- ☐ Between 16 and 30 times
- ☐ More than 30 times (but not every day)
- ☐ I experience arrhythmia on and off every day
- ☐ I have persistent arrhythmia

Question 2

For how long does your arrhythmia <u>usually</u> last?
*(please choose **one** alternative)*

- ☐ Less than1 hour
- ☐ 1 hour – less than 7 hours
- ☐ 7 hours – less than 24 hours
- ☐ 24 hours – less than 2 days
- ☐ 2 days - 7 days
- ☐ More than 7 days

Question 3

What is <u>the longest time</u> for which your arrhythmia lasted?
*(please choose **one** alternative)*

- ☐ Less than 1 hour
- ☐ 1 hour – less than 7 hours
- ☐ 7 hours – less than 24 hours
- ☐ 24 hours – less than 2 days
- ☐ 2 days - 7 days
- ☐ More than 7 days

Question 4

Do you experience any of the following in connection with arrhythmia?
(you can choose more than one alternative)

- ☐ My heart beats fast
- ☐ My heart beats regularly
- ☐ My heart beats irregularly
- ☐ My heart beats harder than usual
- ☐ A feeling that my heart is missing one or more beats
- ☐ Short episodes of arrhythmia lasting less than 1 minute
- ☐ No, I do not experience any of the above

Question 5
Does your arrhythmia occur at specific occasions?

- ☐ No
- ☐ Yes

If ”**Yes**” , please note what occasions

Question 6
What symptoms do you experience in connection with your arrhythmia?

a) Breathlessness during activity

- ☐ Yes, a lot
- ☐ Yes, quite a lot
- ☐ Yes, to a certain extent
- ☐ No

b) Breathlessness even at rest

- ☐ Yes, a lot
- ☐ Yes, quite a lot
- ☐ Yes, to a certain extent
- ☐ No

c) Dizziness

- ☐ Yes, a lot
- ☐ Yes, quite a lot
- ☐ Yes, to a certain extent
- ☐ No

d) Cold sweats
(pale, cold, sweaty)

- ☐ Yes, a lot
- ☐ Yes, quite a lot
- ☐ Yes, to a certain extent
- ☐ No

e) Weakness/fatigue

- ☐ Yes, a lot
- ☐ Yes, quite a lot
- ☐ Yes, to a certain extent
- ☐ No

f) Tiredness

- ☐ Yes, a lot
- ☐ Yes, quite a lot
- ☐ Yes, to a certain extent
- ☐ No

g) Chest pain

- ☐ Yes, a lot
- ☐ Yes, quite a lot
- ☐ Yes, to a certain extent
- ☐ No

h) Pressure/discomfort in chest

- ☐ Yes, a lot
- ☐ Yes, quite a lot
- ☐ Yes, to a certain extent
- ☐ No

i) Worry/anxiety

- ☐ Yes, a lot
- ☐ Yes, quite a lot
- ☐ Yes, to a certain extent
- ☐ No

Question 7
Have you ever come close to fainting in connection with your arrhythmia?

- ☐ No
- ☐ Yes

Question 8

Have you ever fainted in connection with your arrhythmia?

- ☐ No
- ☐ Yes

ASTA part III – Health-related quality of life

This part of the questionnaire deals with how your arrhythmia affects your daily life. Choose the alternative that corresponds the best to your situation.
(If in doubt, please choose the alternative that mostly corresponds to you. If you feel that you cannot determine whether your arrhythmia affects the requested aspect of your life, we recommend that you answer ***no****).*

Question 1

Do you feel unable to work, study or carry out daily activities as you would like to due to your arrhythmia?

- ☐ Yes, a lot
- ☐ Yes, quite a lot
- ☐ Yes, to a certain extent
- ☐ No

Question 2

Do you spend less time with your family/relatives and friends than you would like to due to your arrhythmia?

- ☐ Yes, a lot
- ☐ Yes, quite a lot
- ☐ Yes, to a certain extent
- ☐ No

Question 3

Do you spend less time with acquaintances (people you do not know that well) than you would like to due to your arrhythmia?

- ☐ Yes, a lot
- ☐ Yes, quite a lot
- ☐ Yes, to a certain extent
- ☐ No

Question 4

Do you avoid planning things you would like to do, for instance travelling or leisure activities due to your arrhythmia?

☐ Yes, a lot
☐ Yes, quite a lot
☐ Yes, to a certain extent
☐ No

Question 5

Is your physical ability impaired due to your arrhythmia?

☐ Yes, a lot
☐ Yes, quite a lot
☐ Yes, to a certain extent
☐ No

Question 6

Is your ability to concentrate impaired due to your arrhythmia?

☐ Yes, a lot
☐ Yes, quite a lot
☐ Yes, to a certain extent
☐ No

Question 7

Do you feel dejected or sad due to your arrhythmia?

☐ Yes, a lot
☐ Yes, quite a lot
☐ Yes, to a certain extent
☐ No

Question 8

Do you feel irritated or angry due to your arrhythmia?

- ☐ Yes, a lot
- ☐ Yes, quite a lot
- ☐ Yes, to a certain extent
- ☐ No

Question 9

Do you experience sleep problems due to your arrhythmia?

- ☐ Yes, a lot
- ☐ Yes, quite a lot
- ☐ Yes, to a certain extent
- ☐ No

Question 10

Is your sexual life affected negatively by your arrhythmia?

- ☐ Yes, a lot
- ☐ Yes, quite a lot
- ☐ Yes, to a certain extent
- ☐ No

Question 11

Are you afraid of dying due to your arrhythmia?

- ☐ Yes, a lot
- ☐ Yes, quite a lot
- ☐ Yes, to a certain extent
- ☐ No

Question 12

Has your life situation deteriorated due to your arrhythmia?

- ☐ Yes, a lot
- ☐ Yes, quite a lot
- ☐ Yes, to a certain extent
- ☐ No

Question 13

Do you feel worried that your symptoms will re-occur during the periods when you do not have arrhythmia?

- ☐ Yes, a lot
- ☐ Yes, quite a lot
- ☐ Yes, to a certain extent
- ☐ No

Thank you for taking time to complete this questionnaire!
