# Supplementary figures and images for: Symptoms and health‐related quality of life 5 years after catheter ablation of atrial fibrillation
Source: Clin Cardiol. 2021 Dec 16;45(1):42–50. doi: 10.1002/clc.23752 (PMC8799058; doi:10.1002/clc.23752)

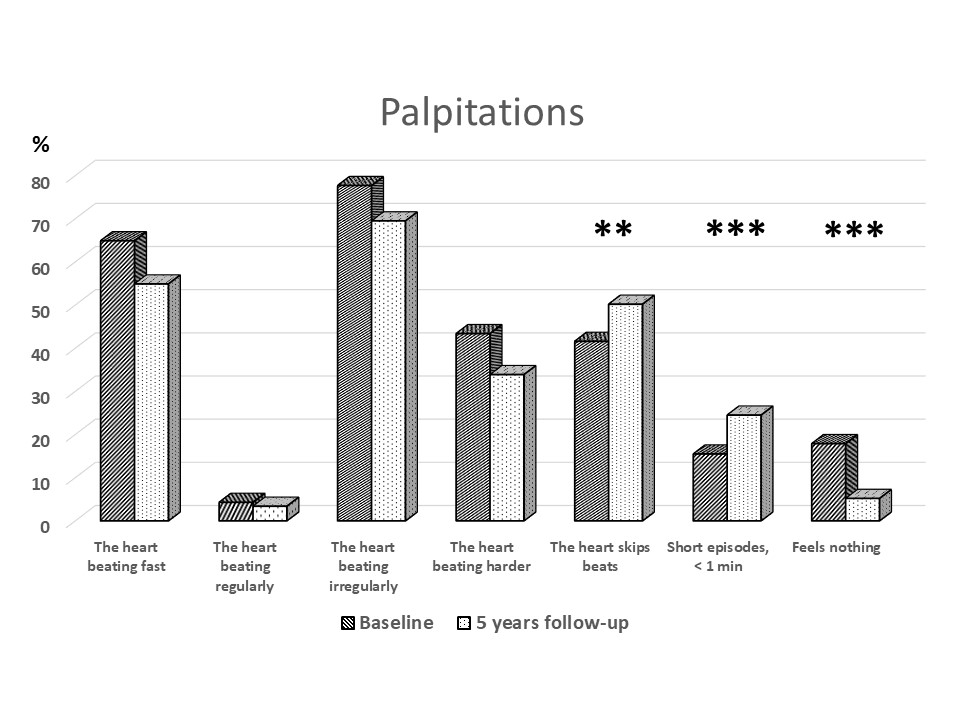

Supplement: Supplementary file 3 — Supporting information. [file CLC-45-42-s001.jpg]
