# Supplementary material for: Symptoms and health‐related quality of life 5 years after catheter ablation of atrial fibrillation
Source: Clin Cardiol. 2021 Dec 16;45(1):42–50. doi: 10.1002/clc.23752 (PMC8799058; doi:10.1002/clc.23752)
Supplement: Supplementary file 6 — Supporting information. [file CLC-45-42-s003.docx]

# Supplementary data and figures

## Supplementary Figure 1a

Legend:

The pie-chart illustrates the ASTA symptom scale score at the five-year follow-up after catheter ablation of atrial fibrillation.

Abbreviation: ASTA: The Arrhythmia-Specific questionnaire in Tachycardia and Arrhythmia.

## Supplementary Figure 1b

Legend:

The pie-chart below illustrates the ASTA HRQoL scale score at the five-year follow-up after catheter ablation of atrial fibrillation.

Abbreviation: ASTA: The Arrhythmia-Specific questionnaire in Tachycardia and Arrhythmia.

## Supplementary figure 2

Answers to the seven questions describing the experienced palpitations in the ASTA questionnaire. A p-value <0.001 is illustrated by ** and a p-value <0.001 by ***.

Figure 2


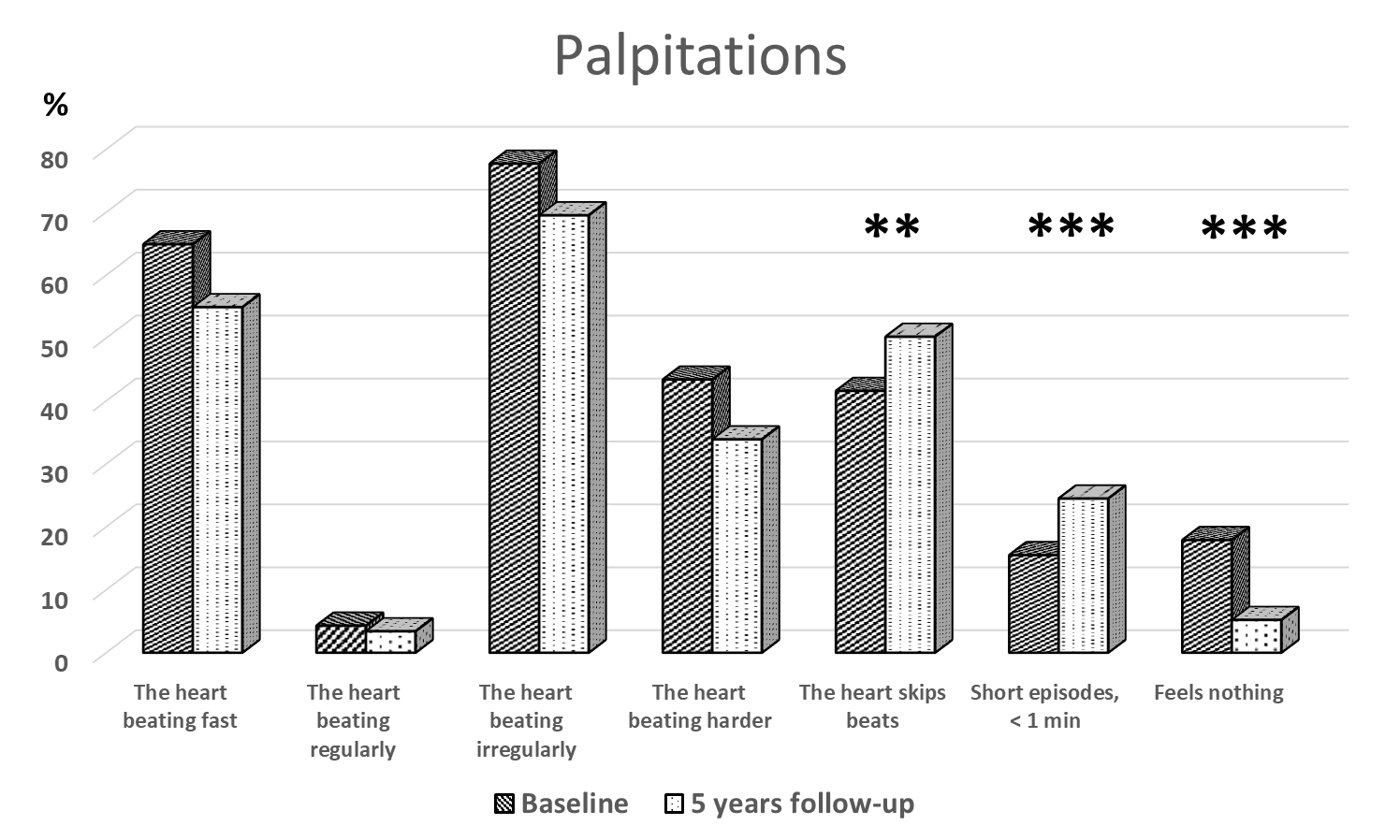


Legend

Results from the nine items in the ASTA symptom scale, where data are presented as Yes or No for each symptom, disregarding intensity.

Footnote: The numbers and percentages presented at the five-year follow-up relate to those patients still experiencing arrhythmia-related symptoms.
